# Suspected Thyroid Storm in a Patient Undergoing Internal Fixation for a Left Mandibular Fracture Under General Anesthesia: A Case Report

**DOI:** 10.7759/cureus.88917

**Published:** 2025-07-28

**Authors:** Mako Domi, Kazushige Koike, Tomohiro Chaki, Takashi Sasaya, Akihiro Miyazaki

**Affiliations:** 1 Department of Oral Surgery, Sapporo Medical University School of Medicine, Sapporo, JPN; 2 Department of Anesthesiology, Sapporo Medical University School of Medicine, Sapporo, JPN

**Keywords:** general anesthesia, hyperthermia, hyperthyroidism, tachycardia, thyroid storm

## Abstract

Thyroid storm is a life-threatening condition in which patients with untreated or poorly controlled thyroid disease experience excessive thyroid hormone action triggered by severe stress, resulting in multiple organ dysfunction. We present a case of suspected thyroid storm during surgery for a left mandibular fracture under general anesthesia. The patient was a 23-year-old male, and a preoperative interview revealed no notable medical history. During open reduction and internal fixation under general anesthesia, the patient developed sinus tachycardia, hypertension, elevated end-tidal carbon dioxide, and persistent hyperthermia. Based on these clinical findings, malignant hyperthermia was initially suspected. However, the absence of myoglobinuria led to the exclusion of this diagnosis, and the surgery was continued. After emerging from general anesthesia, the persistent tachycardia, hypertension, and hyperthermia did not improve; therefore, a physical examination was performed, revealing an enlarged thyroid gland. Furthermore, blood tests showed an abnormally high free triiodothyronine level and an abnormally low thyroid-stimulating hormone level. After referral to the endocrinology department, the patient was diagnosed with hyperthyroidism and a suspected thyroid storm triggered by trauma and surgical stress. In conclusion, thyroid storm should be considered as a differential diagnosis in cases of unexplained refractory tachycardia, hyperthermia, or hypercapnia during anesthesia management.

## Introduction

Thyroid storm is a life-threatening endocrine emergency that occurs when patients with undiagnosed or poorly controlled hyperthyroidism develop severe systemic decompensation in response to acute stressors such as infection, trauma, or surgery [1,2]. Its reported incidence varies, but mortality rates in patients with thyroid storm can be as high as 10-30% in some surveys and up to 20-50% in others, emphasizing the critical need for prompt recognition and management [1,3]. Clinically, thyroid storm can present with high fever, tachycardia, profuse sweating, diarrhea, and altered mental status, although the specific signs can vary widely among individuals [4]. Because of its potentially serious outcomes, guidelines recommend postponing surgical procedures until thyroid function has been optimized [5]. However, when urgent surgery is required, the risk of inducing thyroid storm remains markedly elevated.

In the perioperative period, vigilance is essential to distinguish thyroid storm from malignant hyperthermia or infection, especially in unexplained fever, tachycardia, or hypercapnia [6]. We present the case of a patient who developed suspected intraoperative thyroid storm during open reduction and internal fixation for a mandibular fracture under general anesthesia. This case report highlights the importance of a thorough preoperative evaluation in emergency surgery, including a detailed history and physical examination, enhancing the necessity to consider thyroid storm in the differential diagnosis of perioperative hyperthermia and hemodynamic instability [7].

## Case presentation

**Figure 1:**
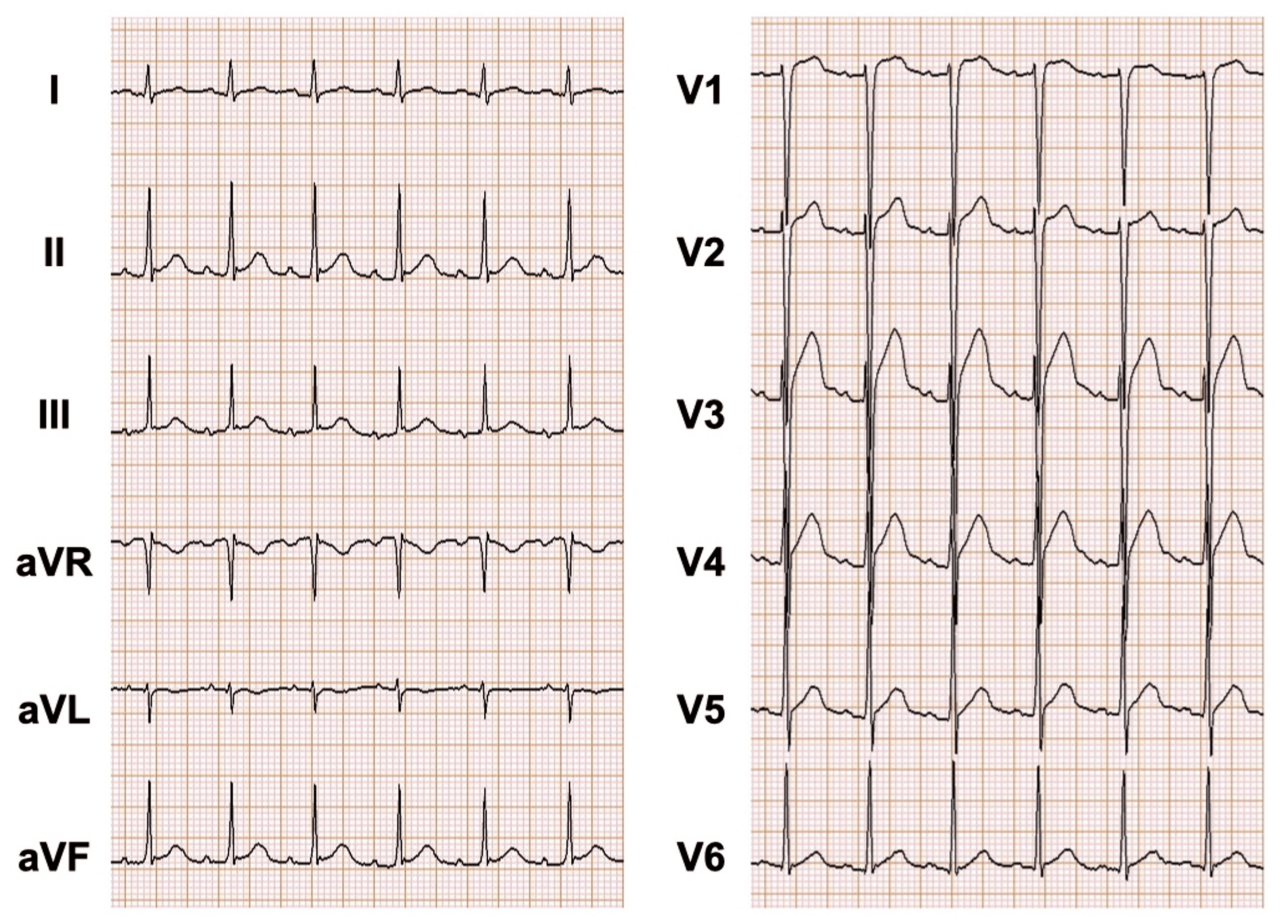
Preoperative electrocardiogram The preoperative electrocardiogram revealed sinus tachycardia with a heart rate of 110 bpm.

A 23-year-old male (height 182.2 cm, weight 53.8 kg) was scheduled for open reduction and internal fixation of a left mandibular fracture under general anesthesia. His medical history included surgery for vesicoureteral reflux at the age of three years. No other significant medical or medication history was reported. Preoperative vital signs were as follows: temperature 36.5 °C, blood pressure 137/77 mmHg, and SpO_2_ 98%. Blood tests revealed elevated alkaline phosphatase (ALP) at 276 U/L, alanine transaminase (ALT) at 471 U/L, γ-glutamyl transpeptidase (γ-GTP) at 90 IU/L, C-reactive protein (CRP) at 0.46 mg/dL, a white blood cell count of 8.8×10^3 ^/μL, and a serum potassium level of 4.6 mEq/L. A preoperative electrocardiogram revealed sinus tachycardia with a resting heart rate of 110 beats/min (Figure 1). The tachycardia was attributed to mild dehydration, as the patient had reduced oral intake due to mandibular pain, and no further cardiac or endocrine evaluation was pursued at that time.

Upon arrival in the operating room, the patient’s heart rate was 95 beats/min, blood pressure 172/101 mmHg, peripheral capillary oxygen saturation (SpO_2_) 95%, and body temperature 36.6°C. General anesthesia was induced with 120 mg of propofol, 50 mg of rocuronium, and a continuous infusion of remifentanil at 0.45 μg/kg/min, followed by tracheal intubation. General anesthesia was maintained with desflurane at 4% (0.61 MAC) under 0.5 L/min oxygen and 1.5 L/min air flow, and remifentanil at 0.3 μg/kg/min.

**Figure 2:**
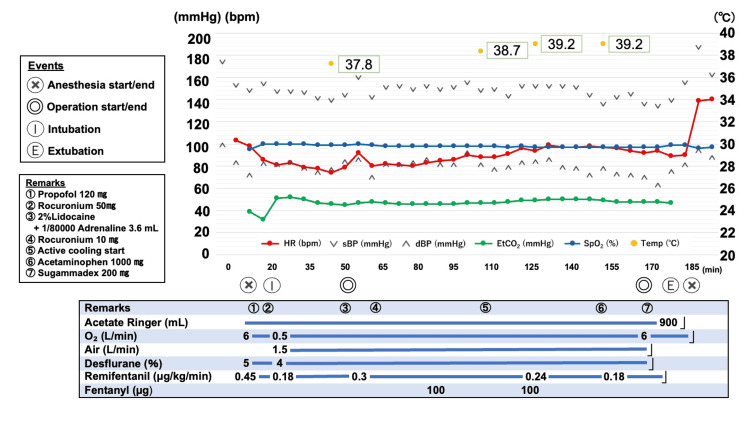
Intraoperative trends in vital signs in a patient with refractory hyperthermia Following the induction of general anesthesia, both body temperature and heart rate gradually increased. Despite active cooling measures, refractory hyperthermia persisted. As no other significant abnormalities were noted aside from hyperthermia and tachycardia, the patient was extubated at the end of surgery and transferred to the general ward. sBP: systolic blood pressure, dBP: diastolic blood pressure, HR: heart rate, EtCO₂: end-tidal CO_2_, SpO_2_: peripheral capillary oxygen saturation, Temp: body temperature

Intraoperatively, the patient developed hyperthermia, with an axillary temperature rising to 39.2°C. He also demonstrated persistent sinus tachycardia with a heart rate of 100 beats/min, systolic blood pressure of 130-150 mmHg, and an end-expiratory partial pressure of carbon dioxide of 50 mmHg; these were refractory to opioid administration (Figure 2). Although malignant hyperthermia was initially considered, it was ruled out based on the absence of rapid temperature escalation, generalized muscle rigidity, or myoglobinuria. Additionally, there was no discoloration of the urine. Even though the hyperthermia persisted despite intraoperative cooling using the Bair Hugger system (3M, Saint Paul, MN, US), the surgery was completed without interruption. The patient remained hemodynamically stable and was extubated at the end of the procedure. Despite noticeable sweating, he was fully awake and transferred to the general ward.

Postoperatively, the patient continued to exhibit persistent tachycardia, with a heart rate of 100 beats/minute, elevated blood pressure (160/100 mmHg), and a body temperature of 37.9 °C. A subsequent neck examination by palpation revealed a diffusely enlarged thyroid gland. Thyroid function tests showed markedly elevated free triiodothyronine (T3) at 20.9 ng/dL (reference range: 0.7-2.0 ng/dL), free thyroxine (T4) at 7.77 μg/dL (reference range: 4.0-13.0 μg/dL), and suppressed thyroid-stimulating hormone (TSH) level of <0.01 μIU/mL (reference range: 0.5 to 5.0 μIU/mL), consistent with severe thyrotoxicosis (Table 1).

**Table 1:** Postoperative thyroid function test The postoperative thyroid function test results showed elevated levels of free triiodothyronine (FT3) and free thyroxine (FT4), along with a suppressed thyroid-stimulating hormone (TSH), suggesting the possibility of thyrotoxicosis. Following seven days of anti-thyroid treatment, both FT3 and FT4 levels decreased.

Parameters	Patient values		Reference range
	After surgery	After 7 days	
Free triiodothyronine (FT3) (ng/dL)	20.9	11.6	0.7-2.0
Free thyroxine (FT4) (µg/dL)	7.77	3.87	4.0-13.0
Thyroid-stimulating hormone (TSH) (µIU/mL)	<0.01	<0.01	0.5-5.0

Following consultation with the endocrinology department, the patient was diagnosed with hyperthyroidism and suspected thyroid storm, likely precipitated by trauma and surgical stress. Treatment was initiated with oral potassium iodide (50 mg/day) and thiamazole (5 mg/day). A more detailed postoperative interview revealed that the patient had experienced symptoms consistent with hyperthyroidism over the past two years, including palpitations, weight loss, excessive sweating, loose stools, and recurrent low-grade fevers. A detailed family history could not be obtained preoperatively. However, postoperative inquiry revealed that the patient’s grandfather had been diagnosed with Graves’ disease and the mother with Hashimoto’s thyroiditis. After seven days of antithyroid therapy, the patient’s free T3 level decreased to 11.6 ng/dL, T4 to 3.87 μg/dL, and TSH remained suppressed at <0.01 μIU/mL (Table 1). His fever resolved by the fourth postoperative day, and he was discharged in stable condition on the eleventh day of hospitalization.

## Discussion

In this case, a thyroid storm was suspected intraoperatively based on sustained tachycardia, hypertension, and elevated end-expiratory partial pressure of carbon dioxide. Postoperative evaluation revealed an enlarged thyroid gland, and laboratory results showed elevated FT3 and suppressed TSH, leading to a diagnosis of hyperthyroidism and suspected thyroid storm. Prompt referral to endocrinology and early therapeutic intervention contributed to an uneventful recovery.

Thyroid storm is a rare but life-threatening endocrine emergency caused by excessive thyroid hormone activity triggered by acute stress, such as trauma, infection, or surgery, in individuals with untreated or poorly controlled hyperthyroidism [8,9]. Despite advances in diagnosis and treatment, thyroid storm continues to carry a high mortality rate, ranging from 10% to 30% [10]. The clinical manifestations are systemic and variable, often including fever, tachycardia, diaphoresis, gastrointestinal symptoms, and neurological disturbances [11]. Common triggers include infection, diabetic ketoacidosis, trauma, radioiodine therapy, surgery, and other acute medical illnesses [4]. In the perioperative setting, the diagnosis can be challenging due to overlapping symptoms with conditions like malignant hyperthermia or sepsis.

The absence of abnormal preoperative findings allowed surgery to proceed under general anesthesia without suspicion of endocrine instability. However, during the surgery, the patient developed unexplained sinus tachycardia and elevated end-tidal carbon dioxide levels. Malignant hyperthermia was considered as one of the differential diagnoses but was ruled out due to the absence of typical findings such as rapid temperature escalation and myoglobinuria. Neuroleptic malignant syndrome was also ruled out in the absence of dopamine antagonist exposure, and systemic infection was deemed unlikely due to normal preoperative inflammatory markers.

The postoperative persistence of tachycardia, hypertension, and fever led to re-evaluation. Physical examination revealed an enlarged thyroid gland, and laboratory studies confirmed thyrotoxicosis. A retrospective medical history revealed longstanding symptoms consistent with hyperthyroidism, including palpitations, weight loss, heat intolerance, and intermittent fevers, along with a family history of autoimmune thyroid disease, which had not been captured during the initial emergency assessment. This highlights the importance of careful preoperative screening, even under time constraints. Additionally, abnormal liver function is a relatively common but often underrecognized finding in patients with hyperthyroidism. In this case, the preoperative elevation of ALT to 471 U/L may have reflected early systemic effects of unrecognized thyrotoxicosis. Such findings, although nonspecific, could serve as important clinical clues when evaluating patients in emergency settings.

The Japan Thyroid Association's diagnostic criteria for thyroid storm require biochemical evidence of thyrotoxicosis and dysfunction in at least one organ system to support diagnosis [11]. The patient also scored 40 points on the Burch-Wartofsky Point Scale - 20 for thermoregulatory dysfunction, 10 for tachycardia, and 10 for a known precipitating event, indicating a high likelihood of thyroid storm [12].

Several key management priorities emerged during this case. First, prompt temperature control was critical. The patient’s body temperature reached 39.2 °C intraoperatively, and elevated temperatures can aggravate thyroid storm-related metabolic stress and neurologic complications. Intravenous acetaminophen was administered preemptively, consistent with guidelines recommending acetaminophen and physical cooling measures such as ice packs or cooling blankets for fever reduction [11]. Second, control of heart rate is essential in mitigating thyroid storm severity. Although the heart rate did not exceed 150 beats per minute in this case, literature suggests that rates above this threshold are associated with increased morbidity and mortality [13-15]. Beta-1 selective beta-blockers or digitalis agents are typically recommended for heart rate control in thyroid storm, particularly in patients with more significant tachyarrhythmias [13]. Although such agents were not used here, earlier recognition could have warranted their consideration. Third, the choice of antipyretic and analgesic agents during the perioperative period requires special attention in the context of suspected thyrotoxicosis, given the potential adverse effects of certain drugs such as nonsteroidal anti-inflammatory drugs (NSAIDs). NSAIDs, especially salicylates, are known to increase circulating free thyroid hormone levels by displacing them from binding proteins, thereby aggravating thyrotoxicosis [4,13]. In this case, acetaminophen was appropriately chosen as the antipyretic agent. Given the sympathetic overactivation seen in thyroid storm, the concurrent use of NSAIDs could have increased the risk of gastrointestinal complications, such as ulcers, further supporting the use of acetaminophen for fever and analgesia in thyrotoxic patients. Additionally, family history is an important factor in the assessment of thyroid dysfunction. Both Graves’ disease and Hashimoto’s thyroiditis have been shown to run in families and exhibit genetic predisposition. Recognizing such history may aid early suspicion and timely diagnosis in similar clinical scenarios.

## Conclusions

We report a case in which intraoperative findings, including tachycardia, hypertension, and elevated end-tidal carbon dioxide, raised suspicion of thyroid storm. Subsequent postoperative evaluation revealed that the patient had an enlarged thyroid gland, leading to the diagnosis of previously unrecognized hyperthyroidism. This case emphasizes the need to consider thyroid storm as a differential diagnosis when unexplained fever, persistent tachycardia, or hypercapnia occurs during the intraoperative period under general anesthesia. It also reinforces the importance of comprehensive preoperative evaluation, including detailed history-taking and physical examination, even in emergency surgical situations.
